# The Correlates of Recoarctation After Repair in Pediatric Patients with Aortic Coarctation: A Real-World Descriptive Study

**DOI:** 10.3390/jcm15187225

**Published:** 2026-09-17

**Authors:** Zhilin Huang, Haolin Wang, Yao Li, Jie Tian, Xin Jin

**Affiliations:** 1 Department of Cardiology Children’s Hospital of Chongqing Medical University, National Clinical Research Center for Children and Adolescents’ Health and Diseases, Ministry of Education Key Laboratory of Child Development and Disorders, Chongqing Municipal Health Commission Key Laboratory of Children’s Vital Organ Development and Diseases, Chongqing 400014, China; 485127@hospital.cqmu.edu.cn; 2 College of Artificial Intelligence Medicine, Chongqing Medical University, Chongqing 400014, China; haolinwang@cqmu.edu.cn; 3 Department of General Practice, Children’s Hospital of Chongqing Medical University, Chongqing 400014, China; 483388@hospital.cqmu.edu.cn; 4 Department of Cardiothoracic Surgery, Children’s Hospital of Chongqing Medical University, Chongqing 400014, China

**Keywords:** coarctation of the aorta, pediatric patients, restenosis, correlated variables, real-world descriptive study

## Abstract

**Background/Objectives**: This retrospective descriptive real-world study aimed to characterize clinical features and factors associated with recoarctation in children with coarctation of the aorta (CoA) and develop a composite metric to quantify postoperative residual aortic obstruction burden. **Methods**: We retrospectively enrolled 126 pediatric CoA patients (98 surgical, 28 interventional) from January 2012 to December 2025. Baseline, perioperative and follow-up data were analyzed descriptively only, without therapeutic comparison. Univariate Cox and 24-month logistic regression screened for potential correlates of recurrent obstruction. Multicollinearity among hemodynamic indicators was assessed, and the Composite Residual Obstruction Score (CROS) derived from principal component analysis (PCA) was developed to mitigate collinearity bias. **Results**: Baseline characteristics differed substantially between treatment groups. Rates of postoperative hypertension and recoarctation were similar. Patients with recoarctation exhibited heavier preoperative and postoperative hemodynamic burden and higher hypertension prevalence. Recoarctation rates varied by CoA subtype, though intergroup differences were not statistically significant (*p* = 0.1518). Hypertension and abnormal hemodynamic indices were associated with higher recoarctation risk. The CROS was generated from four discharge hemodynamic variables; the first principal component explained 49% of variance. The CROS showed strong discriminative ability for 24-month recoarctation (AUC = 0.927; 40.0% for CROS > 28 versus 1.4% for CROS ≤ 28). **Conclusions**: Postoperative hypertension and residual obstruction measured by the CROS were associated with elevated recoarctation risk in children with CoA. As an internally derived exploratory tool from a single-center cohort with limited recoarctation events (n = 20), the CROS integrates collinear hemodynamic indices to facilitate risk evaluation and reduce multicollinearity bias. The CROS cut-off value of 28 is population-specific for the present cohort and should not be interpreted as a well-established universal clinical threshold.

## 1. Introduction

Coarctation of the aorta (CoA) is a common congenital cardiovascular lesion associated with substantial long-term morbidity, including systemic hypertension, left ventricular dysfunction, aortic dissection, and recurrent obstruction, even after initial repair [1,2]. Recoarctation remains one of the most frequent complications after surgical or transcatheter intervention, with reported rates of 5–30% in pediatric populations, representing a major challenge to long-term management [3,4]. Although surgical repair and transcatheter stent implantation are widely used, uniformly recognized treatment selection standards remain controversial, especially for complex CoA accompanied by additional cardiac anomalies [5,6]. Furthermore, the relative importance of demographic, anatomical, and hemodynamic factors in predicting restenosis remains unclear. Inconsistent evidence limits standardized risk stratification and follow-up protocols, critical gaps that hinder personalized care for pediatric patients with CoA.

Recent advances have improved the understanding of CoA pathophysiology, diagnosis, and treatment. Persistent hypertension after repair is driven by aortic wall abnormalities, residual pressure gradients, dysregulation of the renin–angiotensin–aldosterone system, and baroreflex resetting, all of which promote vascular remodeling and restenosis [7,8,9]. A recent meta-analysis confirmed lower restenosis rates with stent implantation compared with balloon angioplasty in children [10]. Nevertheless, high-quality evidence comparing surgical and interventional outcomes in complex CoA remains scarce and limited by small sample sizes [11,12]. The predictive value of key postoperative indices, such as discharge hemodynamic parameters, has not been systematically validated in large pediatric cohorts.

Despite these innovations, critical knowledge gaps remain, which the present descriptive real-world study aims to address. First, studies investigating restenosis correlated risk factors have yielded inconsistent results due to heterogeneous designs and populations [13,14], making it difficult to establish unified clinical reference indicators. Second, the predictive value of early postoperative echocardiographic indices and hypertension has not been firmly validated in large pediatric cohorts, particularly across different CoA subtypes. Third, few studies have systematically compared surgical and interventional outcomes while stratifying by simple versus complex CoA—an important distinction, as complex CoA accounts for most pediatric cases and carries a higher risk of restenosis. Moreover, the combined use of forest plots and the area under the receiver operating characteristic curve (AUC) to evaluate predictive factors for CoA restenosis remains limited, constraining the ability to quantify the discriminatory power of potential risk markers.

Accordingly, this single-center retrospective descriptive study enrolled 126 pediatric CoA patients receiving different clinical managements. We only descriptively analyzed baseline, perioperative and follow-up data without therapeutic comparison, screened correlates of recoarctation via univariate Cox and 24-month logistic regression, and visualized statistical associations with forest plots and AUCs. Given severe multicollinearity among discrete hemodynamic markers that distorts result interpretation, we further established a unified Composite Residual Obstruction Score (CROS) through principal component analysis (PCA) as a core methodological improvement to quantify overall residual aortic obstruction, aiming to reconcile inconsistent prior findings and supply descriptive evidence for standardized long-term surveillance.

## 2. Materials and Methods

### 2.1. Study Patients

A retrospective study was conducted on pediatric patients diagnosed with CoA who underwent surgical or transcatheter interventional repair at Children’s Hospital of Chongqing Medical University, China, between January 2012 and December 2025.

### 2.2. Inclusion Criteria

1. Confirmed CoA diagnosis via transthoracic echocardiography (TTE), contrast-enhanced computed tomography (CT) angiography, or cardiac catheterization;
2. Age ≤ 18 years at the time of intervention;
3. The patient’s principal diagnosis is CoA;
4. Complete perioperative clinical data, hemodynamic parameters, and follow-up records.

A total of 126 eligible patients were enrolled and stratified into the surgical group (n = 98) and transcatheter interventional group (n = 28) according to the treatment modality selected by a multidisciplinary team (cardiologists, cardiac surgeons, and interventional radiologists) based on age, lesion severity, and associated anomalies—ensuring clinically appropriate treatment assignment reflective of real-world practice. Treatment decisions were reached during multidisciplinary team conferences. The team included pediatric cardiologists, pediatric cardiac surgeons, interventional cardiologists, and cardiovascular radiologists. Clinical data, including patient age, body weight, symptoms, transthoracic echocardiography and CT angiography findings, coarctation morphology and severity, aortic arch hypoplasia, concomitant cardiac anomalies, prior repair history and comorbidities were collectively evaluated. Treatment options were discussed, risks and benefits were communicated to patient guardians, and their preferences were considered before a final consensus on surgical or endovascular management was determined.

The Institutional Ethics Committee approved the study, and informed consent was obtained from all patients or their legal guardians before inclusion, in compliance with the Declaration of Helsinki.

### 2.3. Baseline and Perioperative Data Collection

Clinical data, perioperative parameters, and follow-up records were extracted from the hospital electronic medical record system and the picture archiving and communication system by two independent researchers to minimize bias in data extraction. Discrepancies were resolved through consensus with a third senior researcher. For patients with partially incomplete data, missing values were verified by reviewing original admission, discharge and follow-up imaging files. Cases with critical missing core indicators (including preoperative/postoperative echocardiographic hemodynamic gradients, aortic stenosis diameter, and full 24-month follow-up records) were excluded during enrollment to avoid biased risk factor estimation. Minor missing secondary variables (e.g., partial anthropometric data) were not imputed, and only complete paired data were included in statistical comparisons and regression analyses. No multiple imputation or other missing data interpolation methods were applied in this study. The following data were collected and compared between groups:

- Baseline clinical characteristics: age, sex, height, and weight;
- Perioperative parameters: operation time, hospital stay, and preoperative inotropic drug use;
- Hemodynamic indices: Brachial–femoral systolic pressure gradient, Doppler-estimated gradient, peak flow velocity, and stenotic diameter on CT and ultrasound were assessed. Data on associated cardiac anomalies—including aortic arch Z-score, hypoplastic aortic arch (HAA), anomalous pulmonary venous connection, partial anomalous pulmonary venous connection, total anomalous pulmonary venous connection (TAPVC), complete atrioventricular canal defect (CAVC), aortopulmonary window (APW), double outlet right ventricle (DORV), interruption of the aortic arch, vascular ring, bicuspid aortic valve, left superior vena cava, aberrant right subclavian artery, patent foramen ovale (PFO), and bronchial stenosis—were collected and compared between the two treatment groups to identify baseline differences that might influence outcomes.

### 2.4. Brachial–Femoral Systolic Pressure Gradient

Blood pressure was measured using a standard mercury sphygmomanometer, with patients in the supine position, after 10 min of rest. Systolic blood pressure was measured simultaneously at the right brachial artery and right femoral artery, and the gradient was calculated as brachial pressure minus femoral pressure (mmHg), following standardized clinical protocols to ensure measurement consistency. Notably, based on the unified diagnostic thresholds and standardized measurement protocols, all hypertension data were collected during routine follow-up visits that occurred prior to echocardiographic or CT confirmation of restenosis, thereby avoiding circular reasoning between the diagnosis of hypertension and restenosis.

### 2.5. Preoperative Inotropic Drug Use

Recorded as binary variables (yes/no) based on whether patients received inotropic agents (e.g., digoxin and dopamine) within 72 h before surgery, this parameter reflects preoperative hemodynamic instability that may impact perioperative outcomes.

### 2.6. Follow-Up

All patients underwent standardized follow-up at 1, 3, 6, 12 and 24 months postoperatively, including TTE, brachial–femoral systolic gradient testing and clinical blood pressure/symptom assessment for early detection of recoarctation and complications. CT angiography was reserved for patients with TTE findings suggestive of restenosis; CT results served as the definitive diagnostic standard in case of discordant imaging findings given superior aortic lumen resolution. Reintervention for confirmed recoarctation was recorded as a secondary endpoint.

### 2.7. Ultrasound Measurement Reproducibility

All ultrasound measurements were performed offline by a senior physician with 15 years of experience in pediatric cardiac ultrasound using the PACS workstation. Each parameter was measured twice, and the average value was used for statistical analysis. All ultrasound parameter measurements were completed by a single senior physician and entered into the database. To ensure data accuracy, a second researcher randomly verified 20% of the data against the original ultrasound reports.

### 2.8. Definition

Restenosis was defined as a brachial–femoral systolic pressure gradient > 20 mmHg on repeated measurements or a peak flow velocity > 2.5 m/s at the coarctation site on TTE, confirmed by CT angiography when echocardiographic findings were equivocal [15].Patients were stratified into non-restenosis (n = 106) and restenosis (n = 20) groups based on follow-up outcomes.

Postoperative hypertension in this study was diagnosed in accordance with the Chinese Guidelines for the Management of Hypertension. Patients were identified as hypertensive if their systolic blood pressure exceeded (100 + 2 × age) mmHg for boys or (100 + 1.5 × age) mmHg for girls, or if oral antihypertensive drugs were required to sustain normal blood pressure levels.

CoA was classified into simple CoA (no concomitant cardiac defects) and complex CoA (one or more associated anomalies, including ventricular septal defect [VSD], atrial septal defect [ASD], patent ductus arteriosus [PDA], TAPVC, HAA, and bronchial stenosis). Restenosis rates were compared within the complex CoA cohort between surgical and transcatheter interventional subgroups, as well as between simple and complex CoA groups.

### 2.9. Surgical and Interventional Procedures

#### 2.9.1. Surgical Procedures

The surgical approach and repair strategy were comprehensively determined based on children’s concomitant cardiac malformations, preoperative blood pressure, transthoracic echocardiography, CT angiography findings, and guardians’ treatment preferences. Median sternotomy was performed for patients requiring concurrent intracardiac repair with the support of cardiopulmonary bypass (CPB), while left lateral thoracotomy was adopted for those with mild cardiac anomalies that could be corrected minimally without CPB. Four surgical repair techniques were utilized in this cohort, including standard end-to-end anastomosis (EEA), extended end-to-end anastomosis (EEEA), extended aortic arch plasty (ESA, aortic arch reconstruction), and patch aortoplasty (PAP). Intraoperative anastomotic tension served as the core criterion for technique selection. Standard EEA was indicated for short-segment simple aortic coarctation with sufficient aortic tissue for tension-free anastomosis. EEEA was the first-line option for isolated aortic isthmus coarctation, whereas ESA aortic arch reconstruction or PAP patch widening was selectively performed in patients complicated with aortic arch hypoplasia.

#### 2.9.2. Transcatheter Interventional Procedures

All interventional procedures were carried out under general anesthesia via femoral artery access. Intraoperative aortography and invasive pressure monitoring were implemented throughout the operation. Treatment modalities were selected according to preoperative echocardiography, CT angiography results, intraoperative angiographic morphology, and trans-coarctation pressure gradients. Three interventional approaches were adopted, consisting of balloon angioplasty alone, bare metal stent implantation, and covered stent implantation. Balloon angioplasty alone only relies on mechanical dilation of the balloon to release stenotic fibrous rings without implantation of any intravascular supporting device, which yields the shortest operative duration. Bare metal stents maintain vascular patency via metallic frameworks and lack a membrane structure to isolate damaged vessel walls. Covered stents can dilate the narrowed lumen. The attached artificial vascular membrane isolates injured intima and seals dissection tears and aneurysmal cavities, simultaneously relieving coarctation and repairing aortic wall lesions.

#### 2.9.3. Patient Selection Criteria for Surgical Versus Endovascular Repair

Surgical repair was prioritized for infants and younger children with low body weight, patients with significant aortic arch hypoplasia, long-segment aortic coarctation, complex concomitant intracardiac malformations requiring concurrent open cardiac repair, or patients with prior failed endovascular intervention. Endovascular intervention (balloon angioplasty or stent implantation) was mainly applied for older children and adolescents presenting with discrete short-segment native or recurrent aortic coarctation, in the absence of severe aortic arch hypoplasia and complex intracardiac lesions requiring open surgical correction. All therapeutic assignments were based on clinical indications and institutional real-world practice, rather than randomization.

### 2.10. Composite Residual Obstruction Score (CROS) Construction

Isolated hemodynamic indices for post-repair aortic obstruction are collinear and pathologically overlapping. To address this limitation, we developed the CROS via PCA using four discharge metrics: ultrasound gradient, peak velocity, brachial-femoral gradient, reverse-coded luminal diameter. Hypertension was excluded to avoid circular reasoning. PC1 accounting for 49% variance was scaled 0–100 (higher = heavier residual obstruction). The CROS replaced discrete hemodynamic variables in all subsequent analyses to mitigate multicollinearity. Notably, the CROS was derived exclusively from the present cohort and represents an exploratory metric without external validation.

### 2.11. Statistical Analysis

Statistical analyses were performed using the SciPy library (version 1.15) in Python (version 3.13). Continuous variables were presented as the mean ± standard deviation (SD) for normally distributed data (Shapiro–Wilk test, *p* > 0.05) or median (interquartile range, IQR) for non-normally distributed data, and compared between groups using an independent-samples *t*-test or Mann–Whitney U test, respectively. Categorical data were summarized as counts and percentages and analyzed using the χ^2^ test or Fisher’s exact test when expected cell frequencies were <5.

Univariate Cox proportional hazards regression was conducted to identify risk factors for recurrent CoA, with restenosis as the primary endpoint and follow-up duration as the time scale; variables with *p* < 0.1 were included in the forest plot. Univariate logistic regression was applied to assess predictors of 24-month restenosis, with odds ratios (ORs) and 95% confidence intervals (95% CIs) reported. Predictive performance was evaluated using AUC, and variables with an AUC > 0.6 were selected for inclusion in the forest plot. The number of recoarctation events (n = 20) was relatively small, which limited the robustness of multivariable adjustment and may introduce sparse-data bias for hazard ratio estimates, especially for postoperative hypertension with a large observed effect size. Therefore, hazard ratios obtained from univariate analyses should be interpreted as exploratory associations rather than precise quantitative risk estimates. The simplified multivariable Cox model mentioned in the Results Section was prespecified to include only a small set of clinically relevant covariates (age, anatomical complexity, and discharge hemodynamic severity) given the limited event count. The proportional hazards assumption for this Cox model was formally assessed using scaled Schoenfeld residuals, including both global and per-covariate tests computed on rank-transformed event times. No statistically substantial evidence of assumption violation was identified within the present analysis. Results from this multivariable model should also be interpreted with extreme caution due to limited statistical power. A two-tailed *p* < 0.05 was considered statistically significant. 

DeepSeek-V4-Pro was used solely for manuscript language polishing, grammar correction, and readability improvement. No generative AI tools were involved in study design, patient data collection, statistical analysis, or interpretation of research findings. All manuscript content has been fully reviewed, revised, and verified by the authors, who take full responsibility for all scientific statements presented in this article.

## 3. Results

### 3.1. Baseline and Perioperative Characteristics

A total of 126 patients with CoA were enrolled, including 98 in the surgical group and 28 in the transcatheter interventional group (Table 1). The sex distribution was comparable between groups (*p* > 0.05). Patients in the interventional group were significantly older, taller, and heavier, with a significantly shorter follow-up time (all *p* < 0.001). Preoperatively, the transcatheter interventional group had a larger stenotic diameter on CT (*p* = 0.020), a higher brachial–femoral systolic pressure gradient, Doppler-estimated gradient, and peak velocity, and a smaller stenotic diameter on ultrasound (all *p* < 0.001), indicating more severe preoperative obstruction. Notably, the aortic arch Z-score was markedly lower in the surgical cohort (median −3.22 ± 0.55 vs. −0.64 ± 1.09, *p* < 0.001), demonstrating a higher prevalence of aortic arch hypoplasia among surgical patients. At discharge, crude between-group comparisons revealed shorter operative duration, shorter hospital stay and more favorable discharge hemodynamic indices in the transcatheter interventional group (all *p* < 0.001). These unadjusted intergroup differences cannot be interpreted as evidence of superior outcomes with transcatheter intervention, owing to substantial baseline heterogeneity between the two cohorts. All comparisons between surgical and transcatheter groups remain strictly descriptive, and no causal or therapeutic-effect inferences are drawn in this manuscript.

Postoperative hypertension and restenosis rates were similar between groups (all *p* > 0.05). The surgical group had a significantly higher prevalence of HAA, VSD, ASD, PDA, bronchial stenosis, and preoperative inotropic drug use (all *p* < 0.001); no cases of HAA were observed in the transcatheter interventional group.

### 3.2. Classification of Early and Late Postoperative Adverse Events

All adverse events were classified into predefined categories and stratified into early (≤30 days post-procedure) and late (2–24 months follow-up) events, with full data summarized in Table 2.

For early complications within 30 days after repair, no de novo aneurysms, neurological adverse events, paradoxical hypertension, secondary reintervention or all-cause death were observed in either the surgical group (n = 98) or interventional group (n = 28). Early vascular injury was limited to two interventional patients who received covered stent implantation and developed femoral artery pseudoaneurysms. One recovered with manual compression and was discharged; the other underwent thrombin injection followed by pseudoaneurysm resection, with complete restoration of lower limb perfusion before discharge. No early vascular injuries occurred in surgical patients.

For late adverse events occurring 31 days to 24 months after the procedure, no vascular injuries, neurological adverse events, paradoxical hypertension or all-cause deaths were observed in either group. One de novo pseudoaneurysm was detected solely in the interventional group. This patient presented with recurrent coarctation after bare metal stent implantation and recovered after secondary covered stent implantation. No late aneurysms were found in the surgical cohort. Secondary reinterventions were recorded in both groups: 8 in the surgical group (3 after primary balloon angioplasty, 5 after primary stent implantation) and 3 in the interventional group (2 repeat balloon angioplasties, 1 repeat covered stent implantation). All remaining patients with restenosis received regular outpatient follow-up.

### 3.3. Comparisons Between Restenosis and Non-Restenosis Groups

The follow-up duration was significantly shorter in the restenosis group (*p* < 0.001; Table 3). The restenosis group had higher preoperative Doppler gradients and peak velocities (*p* = 0.038 and *p* = 0.013, respectively). At discharge, the restenosis group showed a significantly higher brachial–femoral gradient, Doppler gradient, and flow velocity, and a smaller stenotic diameter (all *p* < 0.001), as well as a significantly higher prevalence of hypertension (*p* < 0.001). Notably, all hypertension data were captured at routine follow-up visits ahead of the time point when restenosis was confirmed by echocardiography or CT, eliminating temporal overlap between the two indicators. No significant differences were found in age, body size, hospital stay, operation time, preoperative imaging indices, sex distribution, preoperative inotropic therapy, aortic arch Z-score or treatment modality (all *p* > 0.05).

### 3.4. Stratified Analysis by CoA Subtype

The simple CoA group comprised 20 patients, all treated with transcatheter intervention, with a restenosis rate of 5.0% (1/20). The complex CoA group included 106 patients: 98 underwent surgery (16.3% restenosis), and 8 received transcatheter intervention (37.5% restenosis). Fisher’s exact test showed no significant difference in restenosis risk between subgroups (OR = 3.0750, *p* = 0.1518). Notably, the sample distribution is extremely unbalanced: there were no surgical cases in the simple CoA cohort, and merely 8 interventional patients were included in the complex CoA subgroup. Such a tiny sample greatly reduces statistical power, and all inter-subgroup comparisons must be interpreted with extreme caution, without generalizing the results to judge treatment advantages. The numerically higher restenosis rate in the interventional subgroup should be interpreted cautiously, given the small sample size (n = 8), which limited statistical power.

### 3.5. Univariable Cox Regression Analysis for Restenosis

**Figure 1:**
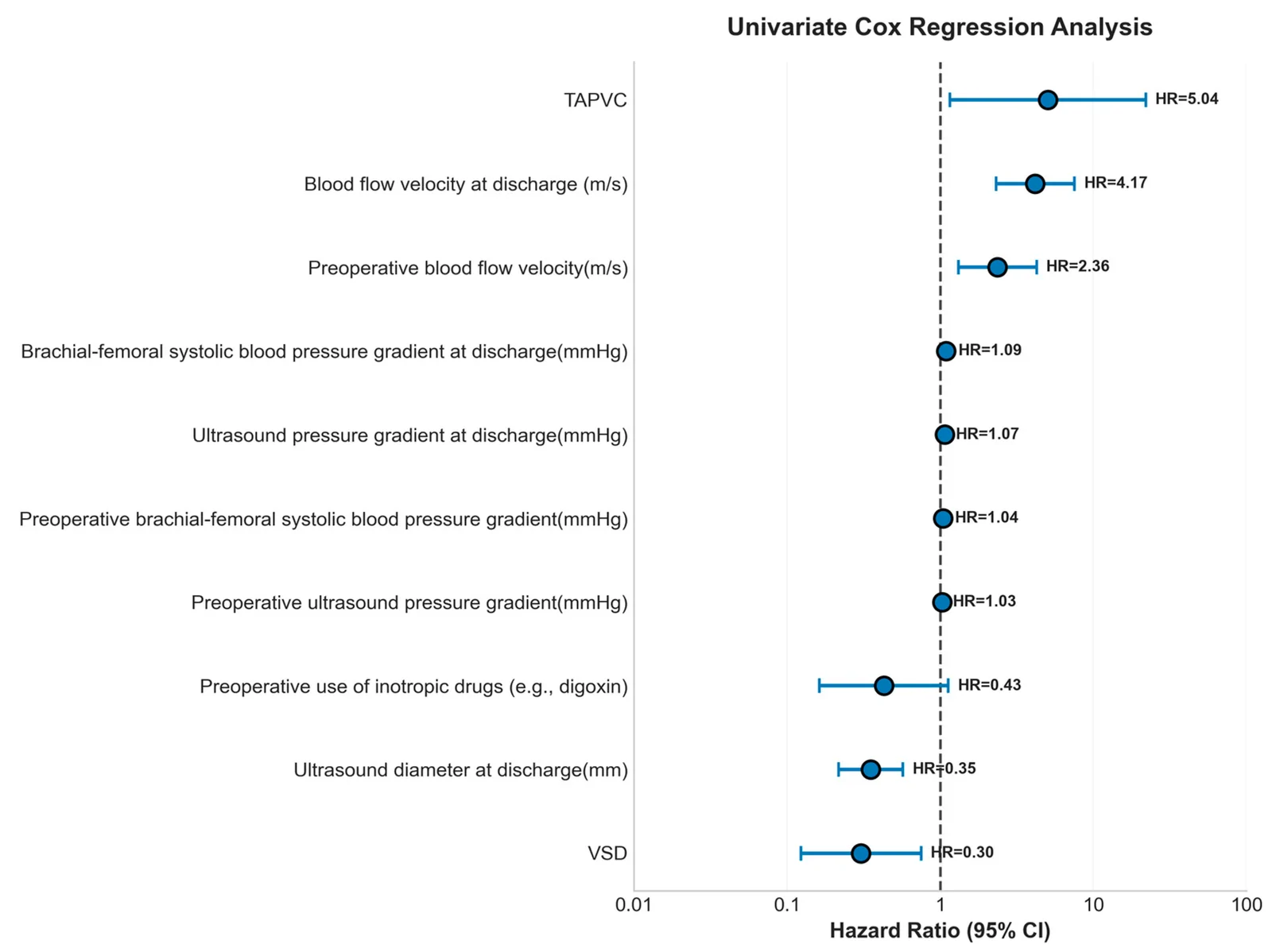
Forest Plot of Univariate Cox Regression Analysis for Restenosis After Coarctation of Aorta Repair.

Univariable Cox proportional hazards regression was performed to identify variables associated with recurrent aortic coarctation, with hazard ratios and corresponding statistical data presented in Table 4 and visualized via forest plots in Figure 1. Multiple postoperative hemodynamic indicators, including hypertension, discharge flow velocity, ultrasound pressure gradient and brachial-femoral systolic gradient, as well as preoperative Doppler hemodynamic parameters and TAPVC, showed positive correlations with recoarctation (all *p* < 0.05). By contrast, larger postoperative aortic luminal diameter and VSD were correlated with a lower risk of recurrence (all *p* < 0.05). Age, sex, body size, preoperative aortic diameter, procedural factors, intervention, aortic arch Z-score and most other anomalies were not significantly associated with restenosis (all *p* > 0.05; Table 4).

After univariate Cox screening of recoarctation correlates, we built a simplified multivariate Cox model adjusted for age, anatomical complexity and postoperative hemodynamic severity. The adjusted hazard ratio forest plot and full statistical outputs are provided in Supplementary Figure S1. Robust multivariate interpretation was limited by only 20 recurrent events (16.0% incidence among 126 patients). 

Of note, the very large hazard estimate for postoperative hypertension should be interpreted cautiously owing to potential sparse-data effects and substantial overlap between postoperative hypertension and subsequent recoarctation; this represents an exploratory association rather than an exact risk quantification.

### 3.6. Multicollinearity Test and Construction of CROS

**Figure 2:**
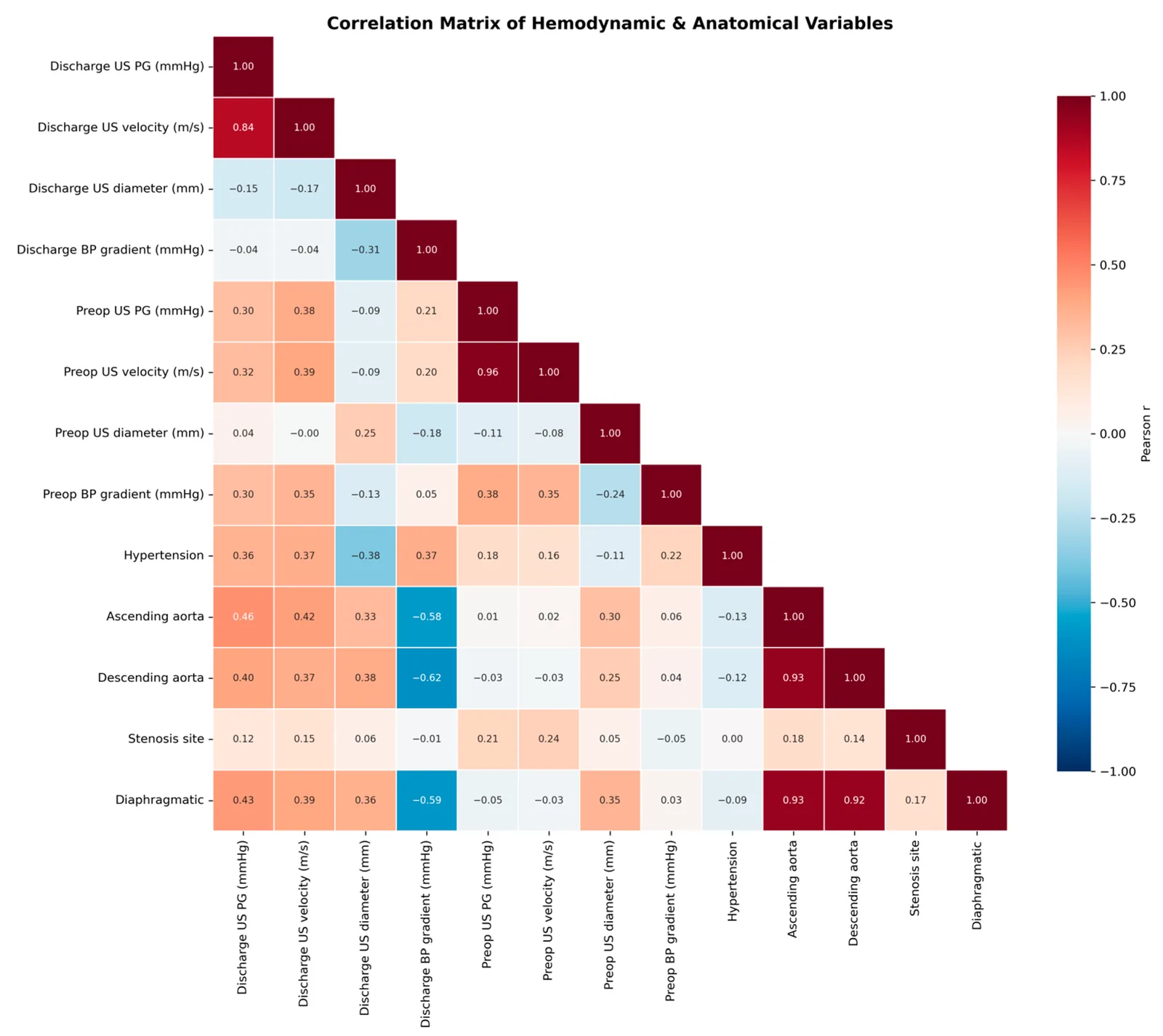
Correlation heatmap of 13 postoperative hemodynamic and aortic anatomical variables, showing notable correlations among discharge hemodynamic indices.

To eliminate bias induced by highly correlated hemodynamic parameters, we performed PCA and variance inflation factor (VIF) assessment covering 13 postoperative hemodynamic and aortic anatomical variables (Figure 2, Supplementary Figure S2). The correlation heatmap (Figure 2) revealed robust pairwise correlations among discharge hemodynamic metrics; notably, the correlation coefficient between ultrasound pressure gradient and peak flow velocity reached 0.75. The VIF distribution visualized in Supplementary Figure S2 further confirmed severe multicollinearity, whereby 11 out of the 13 evaluated variables yielded VIF values exceeding 10. Collectively, these indicators all reflected residual aortic obstruction and were unsuitable for independent inclusion as separate risk predictors.

To address this methodological limitation, PCA was implemented on four core discharge hemodynamic markers (postoperative ultrasound pressure gradient, peak velocity, brachial-femoral pressure gradient, reverse-coded aortic luminal diameter) to generate the novel CROS. The first principal component (PC1) accounted for 49% of the total variance, with all four variables exhibiting positive standardized loadings (Supplementary Figure S3). Postoperative hypertension was excluded from PCA to avoid circular logic, and the derived PCA scores were linearly rescaled to a 0–100 range, where higher CROS values correspond to more severe residual aortic obstruction.

**Figure 3:**
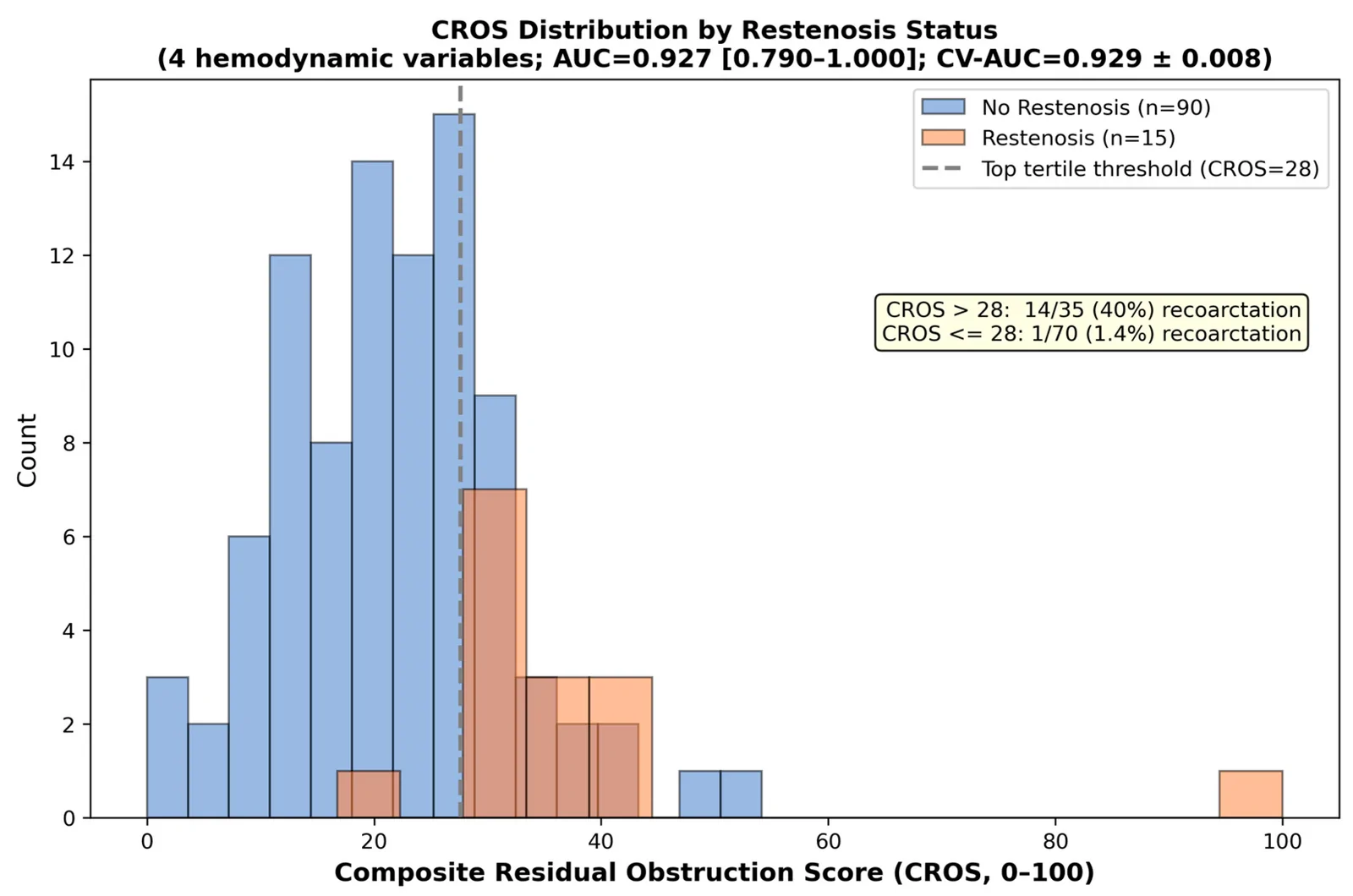
The Distribution of the Composite Residual Obstruction Score (CROS) stratified by the occurrence of 24-month recoarctation. The tertile cut-off value of CROS = 28 was applied for risk stratification of recurrent obstruction.

Receiver operating characteristic (ROC) analysis suggested that the CROS had moderate-to-high discriminatory ability for 24-month recoarctation (AUC = 0.927, 95% CI 0.790–1.000; 5-fold CV-AUC = 0.929 ± 0.008, Figure 3). Using the tertile cut-off of 28, recoarctation risk was 40.0% (14/35) for CROS > 28 versus 1.4% (1/70) for CROS ≤ 28. All subsequent risk analyses adopted this newly developed composite score rather than separate hemodynamic variables to mitigate multicollinearity.

### 3.7. Univariable Logistic Regression Analysis for 24-Month Restenosis

**Figure 4:**
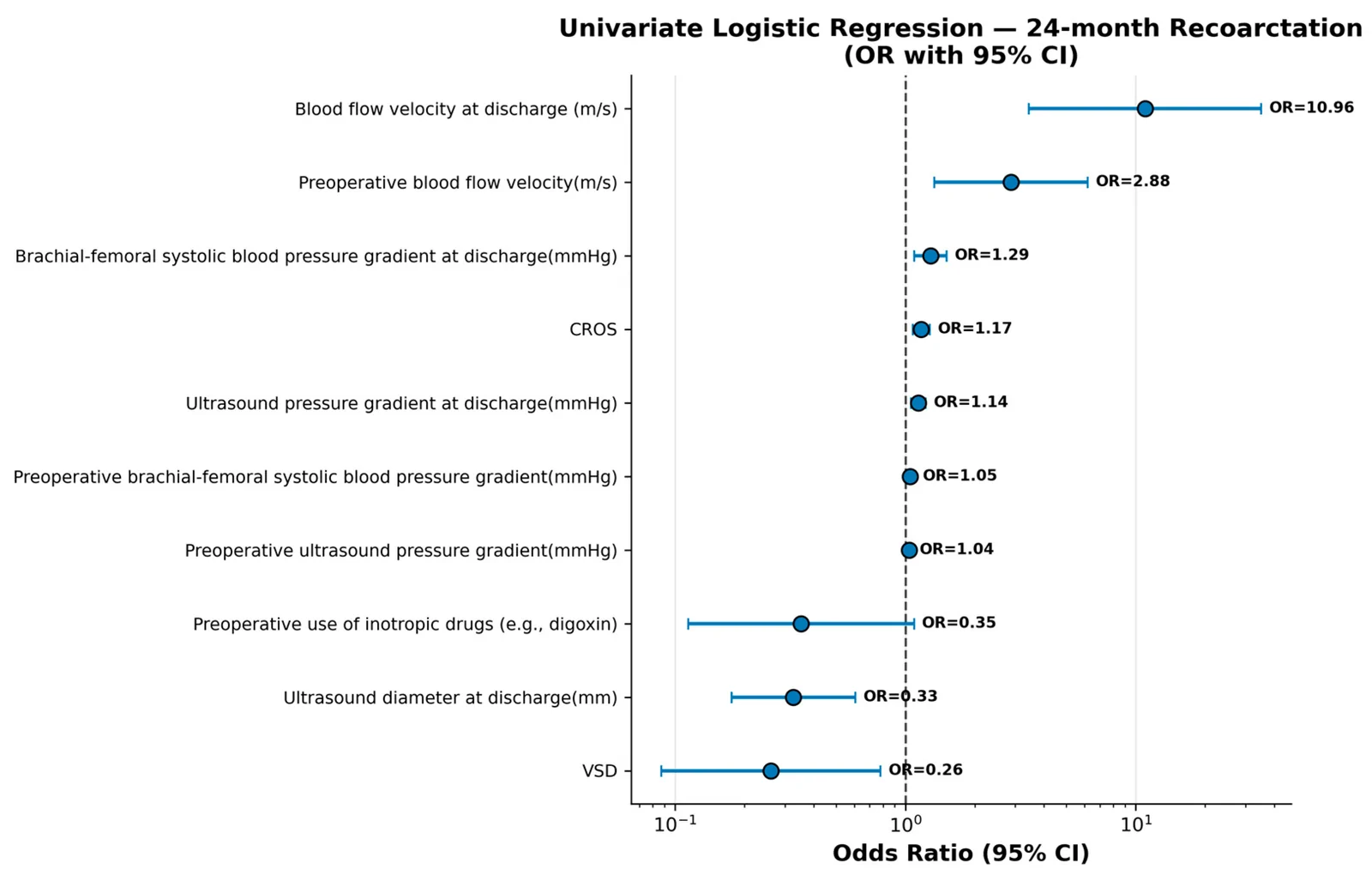
Forest Plot of Univariate Logistic Regression Analysis for 24-Month Restenosis after Aortic Coarctation Repair.

Univariable logistic regression models and corresponding ROC analyses were performed to screen potential predictors of 24-month recurrent aortic coarctation, with all quantitative metrics summarized in Table 5 and regression forest plots visualized in Figure 4. The PCA-derived CROS showed relatively good discriminative performance for recoarctation (OR = 1.173, *p* < 0.001; AUC = 0.9268). Multiple pre- and postoperative hemodynamic parameters, including discharge ultrasound pressure gradient, peak flow velocity, brachial-femoral systolic gradient, and preoperative Doppler indices, were significantly positively correlated with recurrent obstruction. In contrast, larger residual aortic luminal diameter at discharge and ventricular septal defect (VSD) served as protective correlates (all *p* < 0.05). The aortic arch Z-score exhibited no significant association with recurrence and yielded an AUC close to 0.5 (OR = 1.126, *p* = 0.6866). Most demographic, perioperative, and other congenital cardiac variables showed non-significant Wald *p*-values and AUC values near 0.5, with complete ROC optimal cut-off points, sensitivity, and specificity provided in Table 5 for clinical reference.

## 4. Discussion

CoA accounts for approximately 5–8% of all congenital heart defects [16]. Without timely intervention, it can lead to life-threatening complications, including increased left ventricular afterload, refractory hypertension, premature coronary artery disease, and stroke. Traditional surgical procedures, such as end-to-end anastomosis and subclavian artery flap aortoplasty, were once the gold standard for CoA treatment. Since O’Laughlin et al. [17] first described endovascular stent implantation for congenital vascular stenoses including one case of aortic coarctation in 1991, transcatheter stent placement has gradually become a first-line option for adolescents and adults due to its minimal invasiveness, rapid recovery, and low complication rate. With improved success rates of both surgical and transcatheter therapies, recurrent CoA (reCoA) has attracted increasing attention, with a clinical incidence of 5.9–46.6% [3]. Current studies on risk factors for reCoA remain inconsistent, with young age, low body weight, small aortic arch, preoperative hypertension, and trans-coarctation pressure gradient considered potential risk factors [18,19,20].

This study analyzed real-world observational data from 126 children with CoA treated surgically or percutaneously, focusing on descriptive summary of clinical profiles and restenosis-correlated variables rather than inter-group therapeutic comparison. Given the substantial baseline heterogeneity in age, anatomical complexity, and preoperative disease severity, all cross-group comparisons in this study are purely descriptive and cannot be interpreted as evidence of therapeutic superiority or non-inferiority. Baseline descriptive statistics revealed that the transcatheter interventional cohort presented larger body size and more severe preoperative hemodynamic obstruction. Although crude unadjusted records indicated shorter operative and hospital stay durations as well as favorable postoperative hemodynamic relief in this group, such discrepancies stemmed from clinical screening stratification rather than superior therapeutic efficacy, which aligns with previous real-world observational reports [21,22,23]. By contrast, postoperative hypertension and restenosis rates were similar between groups in this unadjusted descriptive comparison, a finding consistent with large registry studies [24]. The surgical group had a higher proportion of complex anomalies and preoperative inotropic support, reflecting more severe clinical conditions, consistent with routine clinical practice [6,25,26].

Stratified analysis by concomitant anomalies showed that the restenosis rate in the simple CoA transcatheter interventional group was 5.0%, similar to previous reports for isolated CoA [12,27]. In complex CoA, although the restenosis rate was numerically higher in the transcatheter interventional subgroup, the difference was not statistically significant, likely due to the small sample size limiting statistical power and because both treatments effectively relieve obstruction, warranting individualized selection. These findings highlight the need for large-scale or multicenter studies to clarify the suitable treatment options for complex CoA.

Subgroup analysis by restenosis status demonstrated that patients with restenosis had higher preoperative hemodynamic load, more severe residual obstruction at discharge, and a significantly higher incidence of hypertension, emphasizing the importance of comprehensive postoperative hemodynamic evaluation and strict blood pressure control during follow-up, consistent with clinical guideline recommendations [28]. The diagnostic workflow for restenosis adopted uniform follow-up imaging schedules and standardized adjudication rules for inconsistent multimodal imaging results to guarantee endpoint reliability. The shorter follow-up time in the restenosis group likely reflects earlier presentation of recurrent obstruction rather than a causal relationship.

Univariate Cox and 24-month logistic regression analyses both showed hypertension was closely correlated with CoA restenosis and had a relatively high AUC value reflecting its statistical association with recurrent obstruction within this cohort. This exploratory observational finding suggests hypertension may serve as a reference indicator for clinical risk stratification of restenosis. Importantly, all hypertension data were collected at routine follow-up visits that preceded imaging-confirmed restenosis, supporting a temporal sequence in which hypertension preceded rather than resulted from recurrent obstruction. This supports systemic hypertension as a major driver of vascular remodeling and recurrent obstruction post-repair. Sustained pressure overload induces vascular smooth muscle cell proliferation and collagen deposition, creating a “hypertension–remodeling–restenosis” cycle that reduces aortic compliance. HAA may also underlie persistent hypertension [7,9]. Elevated discharge flow velocity and pressure gradients, as well as higher preoperative hemodynamic parameters, were associated with increased restenosis risk, while a larger discharge luminal diameter was a consistent protective factor, and these observations suggest residual obstruction is closely correlated with long-term recurrent coarctation within this cohort [29,30,31].

Beyond the associations between obstruction markers and recoarctation, this work offers a notable methodological advancement to address statistical flaws prevalent in prior relevant research. A major methodological innovation of the present study was the development of the CROS composite score to quantify postoperative residual aortic obstruction. Previous pediatric CoA studies separately reported dozens of collinear hemodynamic indices, which inevitably overstated predictive effects and confused clinical interpretation. By integrating four representative postoperative hemodynamic parameters via PCA and excluding hypertension to avoid circularity, the CROS provides a unified, easy-to-apply quantitative tool to comprehensively reflect residual obstruction burden. Satisfactory internal discrimination and cross-validation performance supported its reliability for short-term recoarctation risk stratification. Notably, the CROS and its cut-off value of 28 were entirely derived from this single-center cohort with only 20 recoarctation events; internal cross-validation cannot substitute for independent external-cohort validation, and the 28-point threshold should not be treated as an established clinical cut-off for routine patient management. Unlike discrete single ultrasound measurements, this unified scoring system simplifies risk evaluation and avoids statistical bias caused by multicollinearity, which adds novel methodological evidence to real-world pediatric CoA follow-up research. Before the CROS can be adopted for routine clinical practice, prospective multicenter external validation studies are mandatory. Future investigations need to evaluate model calibration and assess whether the CROS yields incremental predictive value beyond individual conventional hemodynamic indices.

VSD, TAPVC and several other rare associated cardiac anomalies showed statistically exploratory correlations with recoarctation risk in this retrospective cohort. Given small case numbers and multiple potential confounding clinical factors, these associations cannot support definitive mechanistic or causal inference and should be interpreted with great caution. The core clinical take-home message from the present analysis is that residual aortic obstruction identified at hospital discharge acts as an important early warning marker. For children after aortic coarctation repair, strict long-term blood pressure control and intensified serial imaging surveillance are strongly recommended for patients presenting with evidence of residual obstruction.

Notably, age, sex, body weight, operation duration, and most concomitant anomalies were not independently associated with restenosis, consistent with multiple previous studies [18,20]. However, some controversies exist. Burch et al. reported significantly higher recoarctation risk in female patients, while Bacha et al. considered operative weight < 1.5 kg and young age as risk factors [19]. With advances in surgical and transcatheter techniques and perioperative management, the impact of age and weight on postoperative restenosis has weakened significantly compared with earlier eras [32].

At our center, the core principle for reCoA management remains transcatheter intervention as the first-line preferred treatment. Balloon angioplasty is suitable for simple short-segment reCoA without a significant HAA, offering minimal invasiveness and repeatability but carrying risks of restenosis and vascular injury. Stent implantation is indicated for older children with long-segment reCoA, concomitant HAA, or marked elastic recoil after balloon dilation, with lower long-term restenosis rates; staged implantation is required for infants to allow for growth. Surgical treatment is reserved for cases with poor response to transcatheter intervention or complex anatomy, including re-resection and anastomosis, patch aortoplasty, and artificial graft interposition/bypass, tailored to anatomical type and condition. Clinical management should be individualized based on age, weight, anatomy, residual gradient, and previous treatment history. Long-term blood pressure control and regular echocardiographic monitoring are mandatory postoperatively, with intensified follow-up within the first year and long-term vigilance for complications such as aneurysm to improve long-term cardiovascular prognosis.

Several limitations of this study should be noted. First, this was a single-center retrospective observational study with substantial baseline imbalance between surgically and transcatheter-treated patients. Therefore, comparisons between treatment groups remain descriptive, and we cannot draw conclusions regarding the relative efficacy of the two strategies. Second, the CROS was developed internally using data from the present cohort and lacks independent external validation. Accordingly, the CROS should be regarded as an exploratory statistical tool to address multicollinearity rather than a fully validated clinical risk calculator ready for routine clinical application. Its threshold value of 28 is cohort-specific and is not an established universal clinical cut-off. Prospective multicenter external validation including assessment of model calibration and incremental predictive performance compared with conventional hemodynamic indices is required prior to any clinical implementation. Third, the sample size, particularly the transcatheter subgroup, was relatively limited, which may restrict statistical power to detect modest intergroup differences. Fourth, long-term follow-up beyond 24 months was not uniformly available for all participants. In addition, treatment assignment to surgical or endovascular repair was determined by clinical multidisciplinary team assessment instead of randomization. Non-random group allocation introduces inherent selection bias, and baseline clinical differences between groups may influence the observed incidence of recoarctation. Therefore, all inter-group comparisons are strictly descriptive and cannot be used to draw conclusions about comparative therapeutic efficacy.

## 5. Conclusions

Post-discharge hypertension and residual aortic obstruction quantified by the composite CROS descriptive metric are variables correlated with elevated recoarctation risk in this single-center real-world pediatric CoA cohort. This retrospective descriptive study only summarizes real-world clinical distribution characteristics of different clinical management approaches without any comparison of therapeutic effects. We further established the novel exploratory CROS as the core methodological supplement of this work to integrate collinear discrete hemodynamic indicators, which eliminates multicollinearity bias and serves as a unified descriptive tool for recoarctation stratification. The CROS and its 28-point threshold are derived exclusively from our single-center cohort and require prospective multicenter external validation before clinical use. Standardized long-term blood pressure control and serial echocardiographic surveillance are recommended to reduce the observed proportion of recurrent aortic obstruction in children with CoA. 

## Figures and Tables

**Table 1:** Baseline, perioperative, and hemodynamic characteristics between surgical group and interventional groups in patients with aortic coarctation.

Variable	Category	Statistic	Surgical Group	Interventional Group	*p* Value
Sample Size			98	28	
Continuous Variables					
Age (months)		Median (IQR)	2.46 (1.37–4.16)	106.00 (61.00–149.25)	<0.001
Height (cm)		Median (IQR)	54.00 (51.00–58.00)	136.50 (113.25–160.50)	<0.001
Weight (kg)		Median (IQR)	4.20 (3.70–5.10)	27.25 (18.00–44.25)	<0.001
Follow-up time (months)		Median (IQR)	46.20 (33.95–54.60)	12.00 (1.00–24.00)	<0.001
Preoperative CT stenosis diameter (mm)		Mean ± SD	4.34 ± 0.67	4.94 ± 2.13	0.020
Hospital stay (d)		Median (IQR)	20.00 (15.00–24.00)	8.50 (6.00–11.25)	<0.001
Preoperative brachial-femoral systolic blood pressure gradient (mmHg)		Median (IQR)	27.00 (23.00–32.00)	32.00 (26.00–45.00)	0.031
Brachial-femoral systolic blood pressure gradient at discharge (mmHg)		Median (IQR)	8.00 (4.00–13.00)	−6.00 (−12.00–2.00)	<0.001
Preoperative ultrasound pressure gradient (mmHg)		Median (IQR)	36.42 (30.00–48.67)	54.95 (42.15–67.73)	<0.001
Ultrasound pressure gradient at discharge (mmHg)		Median (IQR)	17.00 (12.00–19.00)	26.50 (21.75–34.25)	<0.001
Preoperative ultrasound diameter (mm)		Median (IQR)	3.15 (2.62–3.98)	5.10 (4.10–6.00)	<0.001
Ultrasound diameter at discharge (mm)		Mean ± SD	6.09 ± 1.30	8.48 ± 3.11	<0.001
Preoperative blood flow velocity (m/s)		Median (IQR)	3.05 (2.73–3.49)	3.72 (3.29–4.26)	<0.001
Aortic arch Z-score		Mean ± SD	−3.22 ± 0.55	−0.64 ± 1.09	<0.001
Blood flow velocity at discharge (m/s)		Median (IQR)	1.88 (1.62–2.02)	2.56 (2.33–2.92)	<0.001
Operation time (min)		Median (IQR)	260.00 (230.00–290.00)	71.00 (47.50–90.00)	<0.001
Categorical Variables					
Hypertension		n (%)			1.000
	0		79 (84.0%)	24 (85.7%)	
	1		15 (16.0%)	4 (14.3%)	
Restenosis		n (%)			1.000
	0		82 (83.7%)	24 (85.7%)	
	1		16 (16.3%)	4 (14.3%)	
HAA		n (%)			<0.001
	0		1 (1.0%)	28 (100.0%)	
	1		97 (99.0%)	0 (0.0%)	
VSD		n (%)			<0.001
	0		17 (17.3%)	27 (96.4%)	
	1		81 (82.7%)	1 (3.6%)	
ASD		n (%)			<0.001
	0		28 (28.6%)	24 (85.7%)	
	1		70 (71.4%)	4 (14.3%)	
PDA		n (%)			<0.001
	0		34 (34.7%)	25 (89.3%)	
	1		64 (65.3%)	3 (10.7%)	
PFO		n (%)			0.294
	0		86 (87.8%)	27 (96.4%)	
	1		12 (12.2%)	1 (3.6%)	
DORV		n (%)			1.000
	0		98 (100.0%)	28 (100.0%)	
APW		n (%)			1.000
	0		98 (100.0%)	28 (100.0%)	
CAVC		n (%)			1.000
	0		98 (100.0%)	28 (100.0%)	
TAPVC		n (%)			0.533
	0		96 (98.0%)	27 (96.4%)	
	1		2 (2.0%)	1 (3.6%)	
Vascular ring		n (%)			1.000
	0		98 (100.0%)	28 (100.0%)	
Bicuspid aortic valve		n (%)			0.308
	0		95 (96.9%)	26 (92.9%)	
	1		3 (3.1%)	2 (7.1%)	
Left superior vena cava		n (%)			1.000
	0		91 (92.9%)	26 (92.9%)	
	1		7 (7.1%)	2 (7.1%)	
Aberrant right subclavian artery		n (%)			1.000
	0		96 (98.0%)	28 (100.0%)	
	1		2 (2.0%)	0 (0.0%)	
Bronchial stenosis		n (%)			<0.001
	0		43 (43.9%)	27 (96.4%)	
	1		55 (56.1%)	1 (3.6%)	
Preoperative use of inotropic drugs (e.g., digoxin)		n (%)			<0.001
	0		44 (44.9%)	28 (100.0%)	
	1		54 (55.1%)	0 (0.0%)	
Gender_Male		n (%)			0.477
	0		30 (30.6%)	6 (21.4%)	
	1		68 (69.4%)	22 (78.6%)	
Gender_Female		n (%)			0.477
	0		68 (69.4%)	22 (78.6%)	
	1		30 (30.6%)	6 (21.4%)	

**Table 2:** Summary of Early and Late Postoperative Complications in Surgical and Interventional Cohorts.

Complications Classification	Surgical Group (n = 98)	Intervention Group (n = 28)
Early complications (within 30 days post-procedure)		
Vascular injury	0	2
Newly formed aneurysm	0	0
Neurological adverse events	0	0
Paradoxical hypertension	0	0
Reintervention	0	0
All-cause death	0	0
Late complications (31 days to 24 months post-procedure)		
Vascular injury	0	0
De novo aneurysm	0	1
Neurological adverse events	0	0
Paradoxical hypertension	0	0
Reintervention	8	3
All-cause death	0	0

Notes: Vascular injury included femoral artery pseudoaneurysm, aortic dissection, hemorrhage and vessel occlusion. Neurological adverse events covered cerebral infarction and intracranial hemorrhage. Paradoxical hypertension was defined as sustained elevated blood pressure requiring long-term oral antihypertensive medication. Reintervention indicated secondary invasive procedures performed due to significant recurrent aortic stenosis.

**Table 3:** Comparisons of baseline and perioperative variables between patients with and without postoperative restenosis.

Variable	Category	Statistic	No Restenosis	Restenosis	*p* Value
Sample Size			106	20	
Continuous Variables					
Age (months)		Median (IQR)	3.14 (1.79–8.65)	2.91 (1.40–9.00)	0.492
Height (cm)		Median (IQR)	56.00 (52.00–68.00)	57.50 (52.00–76.50)	0.949
Weight (kg)		Median (IQR)	4.65 (3.80–6.60)	4.45 (4.07–10.00)	0.904
Follow-up time (months)		Median (IQR)	45.70 (30.02–54.12)	18.90 (14.72–23.02)	<0.001
Preoperative CT stenosis diameter (mm)		Median (IQR)	4.46 (3.81–5.00)	4.24 (3.70–4.90)	0.602
Hospital stay (d)		Median (IQR)	17.00 (12.00–22.00)	18.00 (11.00–23.25)	0.952
Preoperative brachial–femoral systolic blood pressure gradient (mmHg)		Median (IQR)	27.00 (23.00–34.00)	30.00 (23.50–39.00)	0.427
Brachial-femoral systolic blood pressure gradient at discharge (mmHg)		Median (IQR)	5.00 (−1.00–11.00)	14.00 (12.25–16.00)	<0.001
Preoperative ultrasound pressure gradient (mmHg)		Median (IQR)	39.85 (31.05–54.42)	51.41 (36.12–66.78)	0.038
Ultrasound pressure gradient at discharge (mmHg)		Median (IQR)	17.00 (12.00–21.00)	21.00 (19.00–28.25)	<0.001
Preoperative ultrasound diameter (mm)		Median (IQR)	3.50 (2.70–4.45)	3.40 (2.85–3.85)	0.779
Ultrasound diameter at discharge (mm)		Median (IQR)	6.20 (5.50–7.35)	5.00 (4.00–5.90)	<0.001
Preoperative blood flow velocity (m/s)		Mean ± SD	3.24 ± 0.65	3.66 ± 0.84	0.013
Blood flow velocity at discharge (m/s)		Median (IQR)	1.97 (1.65–2.17)	2.37 (2.10–2.77)	<0.001
Operation time (min)		Median (IQR)	240.00 (125.00–280.00)	241.50 (127.50–271.25)	0.803
Aortic arch Z-score		Median (IQR)	−3.00 (−3.44-−2.29)	−2.95 (−3.38-−2.37)	0.994
Categorical Variables					
Hypertension		n (%)			<0.001
	0		102 (99.0%)	1 (5.3%)	
	1		1 (1.0%)	18 (94.7%)	
HAA		n (%)			0.779
	0		24 (22.6%)	5 (25.0%)	
	1		82 (77.4%)	15 (75.0%)	
VSD		n (%)			0.133
	0		34 (32.1%)	10 (50.0%)	
	1		72 (67.9%)	10 (50.0%)	
ASD		n (%)			1.000
	0		44 (41.5%)	8 (40.0%)	
	1		62 (58.5%)	12 (60.0%)	
PDA		n (%)			0.330
	0		52 (49.1%)	7 (35.0%)	
	1		54 (50.9%)	13 (65.0%)	
PFO		n (%)			0.691
	0		94 (88.7%)	19 (95.0%)	
	1		12 (11.3%)	1 (5.0%)	
DORV		n (%)			1.000
	0		106 (100.0%)	20 (100.0%)	
APW		n (%)			1.000
	0		106 (100.0%)	20 (100.0%)	
CAVC		n (%)			1.000
	0		106 (100.0%)	20 (100.0%)	
TAPVC		n (%)			0.065
	0		105 (99.1%)	18 (90.0%)	
	1		1 (0.9%)	2 (10.0%)	
Vascular ring		n (%)			1.000
	0		106 (100.0%)	20 (100.0%)	
Bicuspid aortic valve		n (%)			0.585
	0		102 (96.2%)	19 (95.0%)	
	1		4 (3.8%)	1 (5.0%)	
Left superior vena cava		n (%)			0.634
	0		99 (93.4%)	18 (90.0%)	
	1		7 (6.6%)	2 (10.0%)	
Aberrant right subclavian artery		n (%)			1.000
	0		104 (98.1%)	20 (100.0%)	
	1		2 (1.9%)	0 (0.0%)	
Bronchial stenosis		n (%)			1.000
	0		59 (55.7%)	11 (55.0%)	
	1		47 (44.3%)	9 (45.0%)	
Preoperative use of inotropic drugs (e.g., digoxin)		n (%)			0.229
	0		58 (54.7%)	14 (70.0%)	
	1		48 (45.3%)	6 (30.0%)	
Gender_Male		n (%)			0.429
	0		32 (30.2%)	4 (20.0%)	
	1		74 (69.8%)	16 (80.0%)	
Gender_Female		n (%)			0.429
	0		74 (69.8%)	16 (80.0%)	
	1		32 (30.2%)	4 (20.0%)	
Intervention		n (%)			1.000
	0		82 (77.4%)	16 (80.0%)	
	1		24 (22.6%)	4 (20.0%)	

**Table 4:** Univariable Cox Regression of Variables Correlated with Recoarctation After Coarctation of Aorta Repair.

Variable	HR	95% CI Lower Limit	95% CI Upper Limit	*p* Value	log (HR)
Hypertension	171.721	22.284	1323.263	<0.001	5.146
Blood flow velocity at discharge (m/s)	4.173	2.312	7.533	<0.001	1.429
Ultrasound pressure gradient at discharge (mmHg)	1.066	1.038	1.095	<0.001	0.064
Brachial-femoral systolic blood pressure gradient at discharge (mmHg)	1.091	1.050	1.135	<0.001	0.088
Ultrasound diameter at discharge (mm)	0.352	0.217	0.569	<0.001	−1.045
Preoperative blood flow velocity (m/s)	2.364	1.313	4.257	0.004	0.860
Preoperative ultrasound pressure gradient (mmHg)	1.027	1.009	1.047	0.004	0.027
VSD	0.304	0.123	0.749	0.010	−1.192
TAPVC	5.038	1.155	21.979	0.031	1.617
Preoperative brachial–femoral systolic blood pressure gradient (mmHg)	1.039	0.997	1.083	0.068	0.039
Preoperative use of inotropic drugs (e.g., digoxin)	0.428	0.163	1.127	0.086	−0.848
HAA	0.449	0.148	1.368	0.159	−0.800
Gender_Male	1.800	0.597	5.425	0.296	0.588
Gender_Female	0.556	0.184	1.675	0.296	−0.588
Operation time (min)	0.997	0.992	1.002	0.314	−0.003
PDA	1.601	0.606	4.235	0.343	0.471
PFO	0.377	0.050	2.833	0.343	−0.976
Age (months)	0.999	0.986	1.013	0.926	−0.001
Intervention	1.622	0.467	5.628	0.446	0.483
Left superior vena cava	1.760	0.405	7.652	0.451	0.565
Hospital stay (d)	0.983	0.932	1.036	0.513	−0.018
Weight (kg)	0.987	0.942	1.033	0.562	−0.014
Bicuspid aortic valve	1.728	0.230	12.992	0.595	0.547
Bronchial stenosis	0.792	0.322	1.950	0.612	−0.233
Height (cm)	1.003	0.986	1.020	0.736	0.003
Preoperative ultrasound diameter (mm)	0.935	0.632	1.385	0.739	−0.067
Preoperative CT stenosis diameter (mm)	0.945	0.507	1.758	0.857	−0.057
ASD	0.962	0.376	2.463	0.935	−0.039
Aortic arch Z-score	1.056	0.650	1.715	0.825	0.055

**Table 5:** Univariable Logistic Regression of Variables Correlated with 24-Month Recoarctation After Coarctation of Aorta Repair.

Variable	n	Events	OR	OR_95CI_Lower	OR_95CI_Upper	P_Wald	AUC	AUC_95CI_Lower	AUC_95CI_Upper	Optimal_Cut-Off	Sensitivity	Specificity
CROS	94	14	1.173	1.092	1.504	<0.001	0.9268	0.7903	1	0.1605	1	0.85
Ultrasound pressure gradient at discharge (mmHg)	102	17	1.139	1.092	1.309	<0.001	0.8751	0.7179	1	0.1418	0.8824	0.7765
Blood flow velocity at discharge (m/s)	102	17	11.024	5.307	59.83	<0.001	0.8692	0.7089	1	0.1337	0.8235	0.8
Brachial–femoral systolic blood pressure gradient at discharge (mmHg)	99	15	1.286	1.094	2.245	0.0023	0.8397	0.654	1	0.1983	0.9333	0.7143
Ultrasound diameter at discharge (mm)	96	16	0.327	0.123	0.599	<0.001	0.7855	0.5844	0.9867	0.2871	0.5625	0.9125
Preoperative blood flow velocity (m/s)	102	17	2.877	1.354	6.634	0.0068	0.673	0.45	0.896	0.1727	0.5882	0.7294
Preoperative ultrasound pressure gradient (mmHg)	102	17	1.038	1.009	1.07	0.0074	0.6709	0.4476	0.8943	0.1661	0.5882	0.7412
VSD	102	17	0.261	0.078	0.876	0.0163	0.6412	0.4132	0.8692	0.3334	0.4706	0.8118
Preoperative use of inotropic drugs (e.g., digoxin)	102	17	0.353	0.07	1.058	0.0704	0.6235	0.3932	0.8538	0.2353	0.7059	0.5412
Preoperative brachial-femoral systolic blood pressure gradient (mmHg)	101	16	1.049	0.987	1.106	0.048	0.6085	0.3693	0.8476	0.1664	0.5	0.7765
PDA	102	17	2.502	0.8	15.482	0.1336	0.6	0.3671	0.8329	0.2131	0.7647	0.4353
Operation time (min)	102	17	0.997	0.991	1.005	0.3515	0.5696	0.3342	0.8049	0.1724	0.4118	0.7765
TAPVC	102	17	11.249	0.001	45,605.022	0.0545	0.5529	0.3166	0.7893	0.6676	0.1176	0.9882
Preoperative CT stenosis diameter (mm)	97	14	0.745	0.31	1.457	0.4501	0.5478	0.287	0.8085	0.1617	0.5	0.747
Gender_Female	102	17	0.626	0.094	2	0.448	0.5471	0.3104	0.7837	0.1857	0.7647	0.3294
Gender_Male	102	17	1.596	0.5	10.644	0.448	0.5471	0.3104	0.7837	0.1857	0.7647	0.3294
HAA	102	17	0.419	0.097	446.607	0.245	0.5471	0.3104	0.7837	0.3	0.1765	0.9176
Age (months)	102	17	0.997	0.814	1.017	0.8036	0.5412	0.3043	0.7781	0.1699	0.4706	0.7529
ASD	102	17	0.702	0.231	2.698	0.5151	0.5412	0.3043	0.7781	0.1999	0.4118	0.6706
Preoperative ultrasound diameter (mm)	101	17	0.858	0.481	1.309	0.5383	0.5396	0.3026	0.7765	0.1605	0.8235	0.3571
Height (cm)	102	17	1.004	0.976	1.029	0.7586	0.5388	0.3018	0.7757	0.1695	0.3529	0.8471
PFO	102	17	0.421	0	2.13	0.4225	0.5353	0.2982	0.7724	0.1778	0.9412	0.1294
Bronchial stenosis	102	17	0.79	0.248	2.401	0.6581	0.5294	0.2921	0.7667	0.1837	0.5294	0.5294
Left superior vena cava	102	17	2.136	0.001	11.724	0.3907	0.5294	0.2921	0.7667	0.2859	0.1176	0.9412
Intervention	102	17	1.486	0.001	7.152	0.6414	0.5176	0.2801	0.7552	0.2223	0.1176	0.9176
Bicuspid aortic valve	102	17	1.711	0	26.432	0.6518	0.5118	0.2741	0.7494	0.2503	0.0588	0.9647
Aberrant right subclavian artery	102	17	0.002	0	1	0.9762	0.5118	0.2741	0.7494	0.17	1	0.0235
Recurrent CoA	102	17	1.002	0.141	1986.715	0.826	0.5	0.2623	0.7377	inf	0	1
Hospital stay (d)	102	17	0.994	0.935	1.048	0.8372	0.4962	0.2585	0.7339	0.1612	0.9412	0.1765
Weight (kg)	102	17	0.993	0.874	1.096	0.048	0.4668	0.2296	0.7039	0.1699	0.1765	0.9412
Aortic arch Z-score	100	15	1.126	0.621	2.005	0.6866	0.5533	0.3017	0.8049	0.1486	0.6667	0.5529

## Data Availability

All data generated during the current study are accessible in the Supplementary Materials. This file comprises the complete set of extracted variables and the analytical coding framework required to reproduce the comparative results presented herein.

## Supplementary Materials

The following supporting information can be downloaded at: https://www.mdpi.com/article/10.3390/jcm15187225/s1, Figure S1: Forest plot of multivariate Cox regression adjusted for age, anatomical complexity and postoperative hemodynamic severity for predictors of 24-month recurrent aortic coarctation. Full statistical results are presented alongside hazard ratios and corresponding 95% CIs; Figure S2: Correlation heatmap of 13 postoperative hemodynamic and aortic anatomical variables, showing strong intercorrelations among discharge hemodynamic indices; Figure S3: Standardized loadings of four discharge hemodynamic variables on the first principal component (PC1) for constructing the Composite Residual Obstruction Score (CROS). PC1 explained 49% of total variance and represented a single latent dimension of residual aortic obstruction.

## Abbreviations

The following abbreviations are used in this manuscript:

CoA	coarctation of the aorta
CROS	Composite Residual Obstruction Score
PCA	principal component analysis
VIF	variance inflation factor
AUC	the area under the receiver operating characteristic curve
TTE	transthoracic echocardiography
CT	computed tomography
HAA	hypoplastic aortic arch
TAPVC	total anomalous pulmonary venous connection
CAVC	complete atrioventricular canal defect
APW	aortopulmonary window
DORV	double outlet right ventricle
PFO	patent foramen ovale
VSD	ventricular septal defect
ASD	atrial septal defect
PDA	patent ductus arteriosus
EEA	end-to-end anastomosis
EEEA	extended end-to-end anastomosis
