# Supplementary material for: The Correlates of Recoarctation After Repair in Pediatric Patients with Aortic Coarctation: A Real-World Descriptive Study
Source: J Clin Med. 2026 Sep 17;15(18):7225. doi: 10.3390/jcm15187225 (PMC13607068; doi:10.3390/jcm15187225)
Supplement: Supplementary file 1 [file jcm-15-07225-s001.zip › jcm-4494982-supplementary.pdf]

Supplementary Information for 《Correlates of Recoarctation After Repair in Pediatric Patients with Aortic Coarctation: A Real-World Descriptive Study》

Supplementary Figures

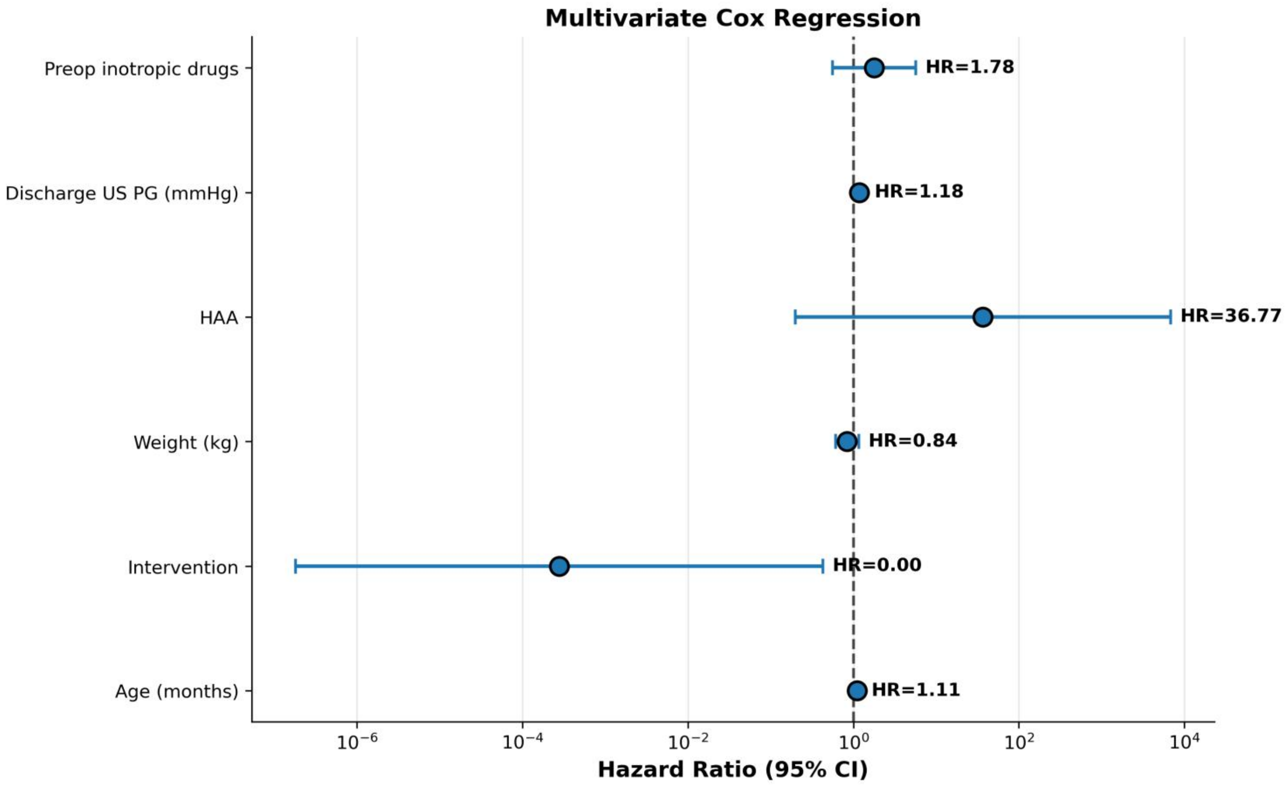

Supplementary Figure S1. Forest plot of multivariate Cox regression adjusted for age, anatomical complexity and postoperative hemodynamic severity for predictors of 24-month recurrent aortic coarctation. Full statistical results are presented alongside hazard ratios and corresponding 95% CIs.

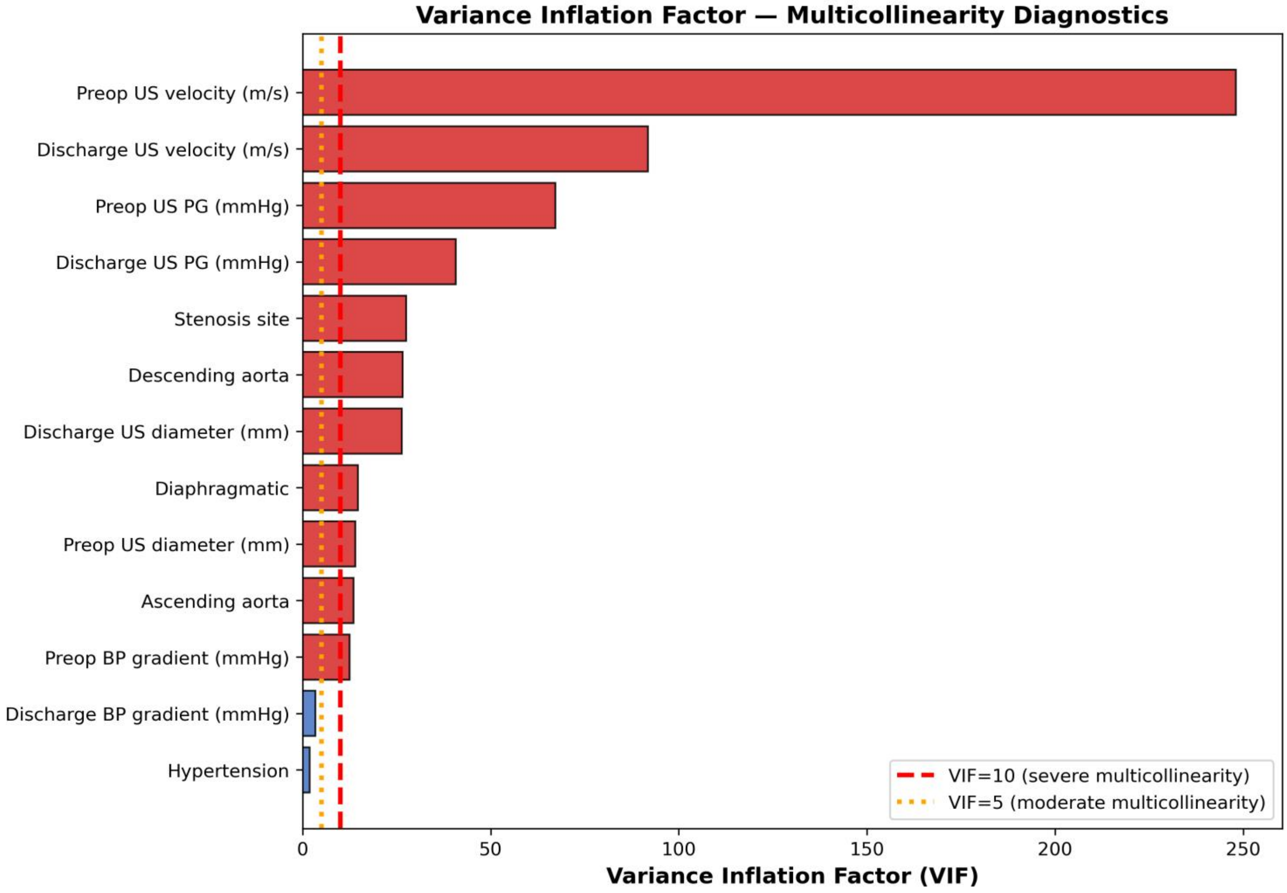

Supplementary Figure S2. Correlation heatmap of 13 postoperative hemodynamic and aortic anatomical variables, showing strong intercorrelations among discharge hemodynamic indices.

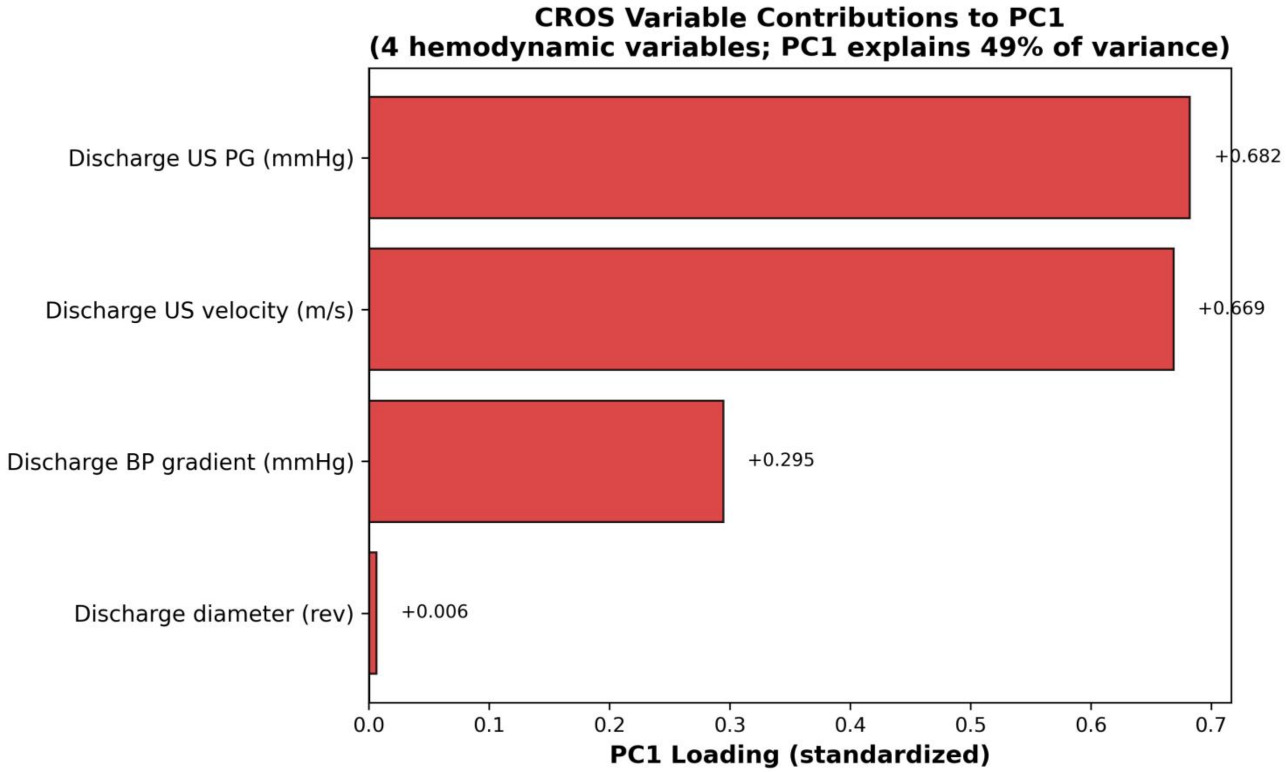

Supplementary Figure S3. Standardized loadings of four discharge hemodynamic variables on the first principal component (PC1) for constructing the Composite Residual Obstruction Score (CROS). PC1 explained 49% of total variance and represented a single latent dimension of residual aortic obstruction.
